# Effect of dietary inclusion of rumen-protected flaxseed oil *(Linum usitatissimum)* on intake, ingestive behavior, diet particle selectivity, digestibility, nitrogen balance, and blood parameters of lambs

**DOI:** 10.1007/s11250-026-05406-x

**Published:** 2026-10-10

**Authors:** Sonel Gilles, José Morais Pereira Filho, Lucas de Souza Barros, Kevily Henrique de Oliveira Soares de Lucena, Romilda Rodrigues do Nascimento, Gildenia Araújo Pereira, Ronaldo Lopes Oliveira, Rui José Branquinho Bessa, Antônio Fernando de Melo Vaz, Leilson Rocha Bezerra

**Affiliations:** 1 Graduate Program in Animal Science and Health, Federal University of Campina Grande, Patos, Paraíba, 58708110 Brazil; 2 Department of Animal Science, Federal University of Bahia, Salvador, Bahia 40170155 Brazil; 3 Laboratório Associado para Ciência Animal e Veterinária (AL4AnimalS), Faculdade de Medicina Veterinária, CIISA – Centro de Investigação Interdisciplinar em Sanidade Animal, Universidade de Lisboa, Av. da Universidade Técnica, Lisboa, 1300-477 Portugal

**Keywords:** Flaxseed oil, Physically effective fiber, Encapsulation, Oil, Ruminants

## Abstract

This study evaluated the effects of rumen-protected flaxseed oil (RPFO; 30% flaxseed free oil and 70% carnauba wax) on intake, digestibility, ingestive behavior, nitrogen balance, and blood parameters in crossbred male lambs (Santa Inês × mixed breed). Fifty lambs (20.5 ± 3.0 kg; five months old) were assigned to a completely randomized design with five treatments: control, 4% and 8% free oil, and 4% and 8% RPFO (*n* = 10 per treatment). Flaxseed free oil inclusion, regardless of form, significantly affected intake, digestibility, feeding behavior, and nitrogen metabolism (*P* < 0.05). Lambs fed 8% free oil showed the lowest (*P* = 0.009) dry matter intake (DMI), whereas 4% RPFO resulted in the highest DMI. Crude protein and neutral detergent fiber corrected for ash and protein (_a_NDFom) intakes decreased (*P* < 0.05) with 8% free oil, while control, 4% free oil, and 8% RPFO showed higher intake and feed efficiency. Average daily gain was lowest with 8% free oil (169 g/day). Free oil reduced rumination time and fiber eating efficiency (*P* < 0.01), while RPFO (4%) improved chewing activity and fiber utilization. Animals receiving free oil showed greater rejection of coarse particles (*P* < 0.01), whereas RPFO maintained more uniform particle selection and physically effective fiber intake. Nutrient digestibility generally decreased with oil inclusion, especially with RPFO at 4% (*P* < 0.05). Nitrogen intake was reduced with 8% free oil and 4% RPFO, while urinary N excretion increased with RPFO (*P* < 0.001), resulting in lower N retention efficiency at 4% RPFO. Blood biochemical parameters were not affected (*P* > 0.05). The RPFO mitigated negative effects of unsaturated lipids on rumen function, whereas high free oil reduced intake and performance. However, RPFO levels tested did not improve overall efficiency, indicating the need for further optimization.

## Introduction

Vegetable lipid supplementation has become an important nutritional strategy in ruminant production because it increases the energy concentration of diets and may influence metabolic responses, animal performance, and the fatty acid composition of meat and milk (Hiller 2014; Dewanckele et al. 2020). Among plant-derived lipid sources, flaxseed oil (*Linum usitatissimum*) is of particular interest because of its high concentration of α-linolenic acid (C18:3 *n* − 3), a precursor of long-chain *n* − 3 fatty acids. Consequently, its inclusion in ruminant diets has been explored as a means of improving the lipid profile and nutritional value of animal-derived foods (Bas et al. 2007; Perkins et al. 2025).

The nutritional benefits of unsaturated oils, however, may be constrained by their effects within the rumen. Excessive amounts of free lipids, particularly when total dietary fat exceeds approximately 6–7% of dry matter, can interfere with ruminal fermentation, suppress fibrolytic microbial populations, modify biohydrogenation pathways, and ultimately decrease feed intake and fiber digestion (Dewanckele et al. 2020; Toral et al. 2024). In addition, extensive ruminal biohydrogenation substantially reduces the proportion of dietary PUFA that reaches the small intestine in its original form. More than 90% of ingested PUFA may undergo biohydrogenation in the rumen, thereby limiting post-ruminal availability and the efficiency with which supplemental unsaturated fatty acids can be incorporated into animal tissues and products (Hess et al. 2008; Wardiman et al. 2024).

Lipid protection technologies represent an alternative approach to overcoming these limitations. By reducing contact between unsaturated fatty acids and ruminal microorganisms, protective matrices may attenuate the negative effects of free oils on the ruminal environment while increasing the amount of intact fatty acids available for intestinal absorption (Carvalho et al. 2019; de Medeiros et al. 2019; de Lucena et al. 2024; Bezerra et al. 2025). Plant-derived waxes have attracted particular interest as encapsulating materials because of their physical stability and relatively low susceptibility to degradation in the rumen (de Freitas et al. 2019; Silva et al. 2023).

Carnauba wax, obtained from *Copernicia prunifera*, has characteristics that make it particularly suitable for lipid protection. This biomaterial is naturally hydrophobic, has a high melting point, and exhibits considerable resistance to chemical and biological degradation (de Freitas et al. 2019). Moreover, carnauba palm is native to Brazil, and its wax is commercially available and extensively used by the food, pharmaceutical, and cosmetic industries, which favors its accessibility for the development of feed technologies (Silva et al. 2025). When used as an encapsulating matrix, these properties may reduce the exposure of PUFA to the ruminal environment and potentially preserve their nutritional functionality without eliciting the digestive disturbances commonly associated with free unsaturated oils.

Nevertheless, the effectiveness of rumen-protected lipids cannot be inferred solely from the characteristics of the encapsulating material. Animal responses may vary according to the amount and type of lipid supplied, the composition of the basal diet, and the extent to which the protective matrix remains stable within the rumen (Dewanckele et al. 2020; INCT 2024). Furthermore, information regarding the effects of protected flaxseed oil on feeding behavior, nutrient utilization, nitrogen metabolism, feed particle selection, and systemic metabolic responses in growing lambs remains limited. Direct comparison between free and protected forms of the same lipid source is therefore important for determining whether protection modifies the digestive and metabolic responses associated with flaxseed oil supplementation.

Accordingly, this study evaluated the effects of free and rumen-protected flaxseed oil on feed intake, apparent nutrient digestibility, feeding behavior, nitrogen balance, feed particle selection, and blood biochemical parameters in growing lambs. We hypothesized that protecting flaxseed oil within a lipid matrix would reduce the adverse digestive responses associated with exposure to free unsaturated oil in the rumen, thereby maintaining feed intake and nutrient digestibility while modifying metabolic responses to lipid supplementation. By integrating digestive, behavioral, nitrogen-utilization, and blood measurements, the study aimed to determine whether rumen protection provides a nutritional advantage over supplementation with unprotected flaxseed oil.

## Materials and methods

### Ethical procedures

All experimental procedures involving animals were conducted in compliance with the institutional standards for animal care and experimentation of the Federal University of Campina Grande (UFCG), Paraíba, Brazil, and were approved by its Animal Ethics Committee under Protocol CEUA No. 02/2024.

#### Rumen-protected flaxseed oil (RPFO) production and characterization

Rumen-protected flaxseed oil (RPFO) was prepared by melt emulsification using carnauba wax as the protective matrix and flaxseed oil (*Linum usitatissimum*) as the lipid core. The procedure was based on the emulsification–fusion approach described by de Medeiros et al. (2019), with adaptations for vegetable oils according to Lima et al. (2023) and Feitosa et al. (2024). Flaxseed oil and carnauba wax were combined at a 30:70 core-to-matrix ratio (w/w, DM basis). The flaxseed oil was obtained commercially (Giroil^®^, Entre-Ijuís, Rio Grande do Sul, Brazil).

Type 1 (prime yellow) carnauba wax (Ageocera^®^, Parnaíba, Piauí, Brazil) was used as the encapsulating material. This commercial grade is characterized by a light yellow-to-golden appearance and a low impurity content (≤ 0.5%). According to the manufacturer’s specifications, the wax had a melting range of 80–86 °C, density of 0.97–0.99 g/cm³ at 25 °C, acid value of 2.0–10.0 mg KOH/g, saponification value of 75–95 mg KOH/g, ester value of 70–90 mg KOH/g, iodine value of 8–12 g I₂/100 g, and refractive index of 1.455–1.460 at 75 °C. The wax contained 980 g DM/kg and 97.5 g ether extract/kg DM and consisted predominantly of high-molecular-weight aliphatic esters and long-chain fatty alcohols, with smaller proportions of hydrocarbons, free fatty acids, and cinnamic acid derivatives.

To prepare the RPFO, carnauba wax was heated in a water bath to 90 °C until completely melted. Flaxseed oil was then incorporated into the molten wax under continuous stirring to obtain a homogeneous lipid phase. This mixture was transferred into distilled water previously heated to 90 °C and containing polysorbate 80 (2.5%, v/v) as the emulsifier. The resulting dispersion was stirred magnetically at 500 rpm for 3 min. Rapid cooling was subsequently induced with water at 4 °C, promoting solidification of the lipid particles. After solidification, the particles were recovered by sieving and washed to remove residual surfactant, following the general procedure described by de Medeiros et al. (2019).

Encapsulation efficiency was estimated from the proportion of flaxseed oil retained within the particles relative to the amount initially incorporated into the formulation. Oil concentration was determined by UV–Vis spectrophotometry at 450 nm using a calibration curve specifically prepared for flaxseed oil, based on the analytical procedure described by de Medeiros et al. (2019). Production yield was calculated from the mass of material recovered after encapsulation relative to the initial mass of flaxseed oil and carnauba wax used in the process. The resulting RPFO showed a production yield of 94.0% and an encapsulation efficiency of 92.43%.

**Fig. 1:**
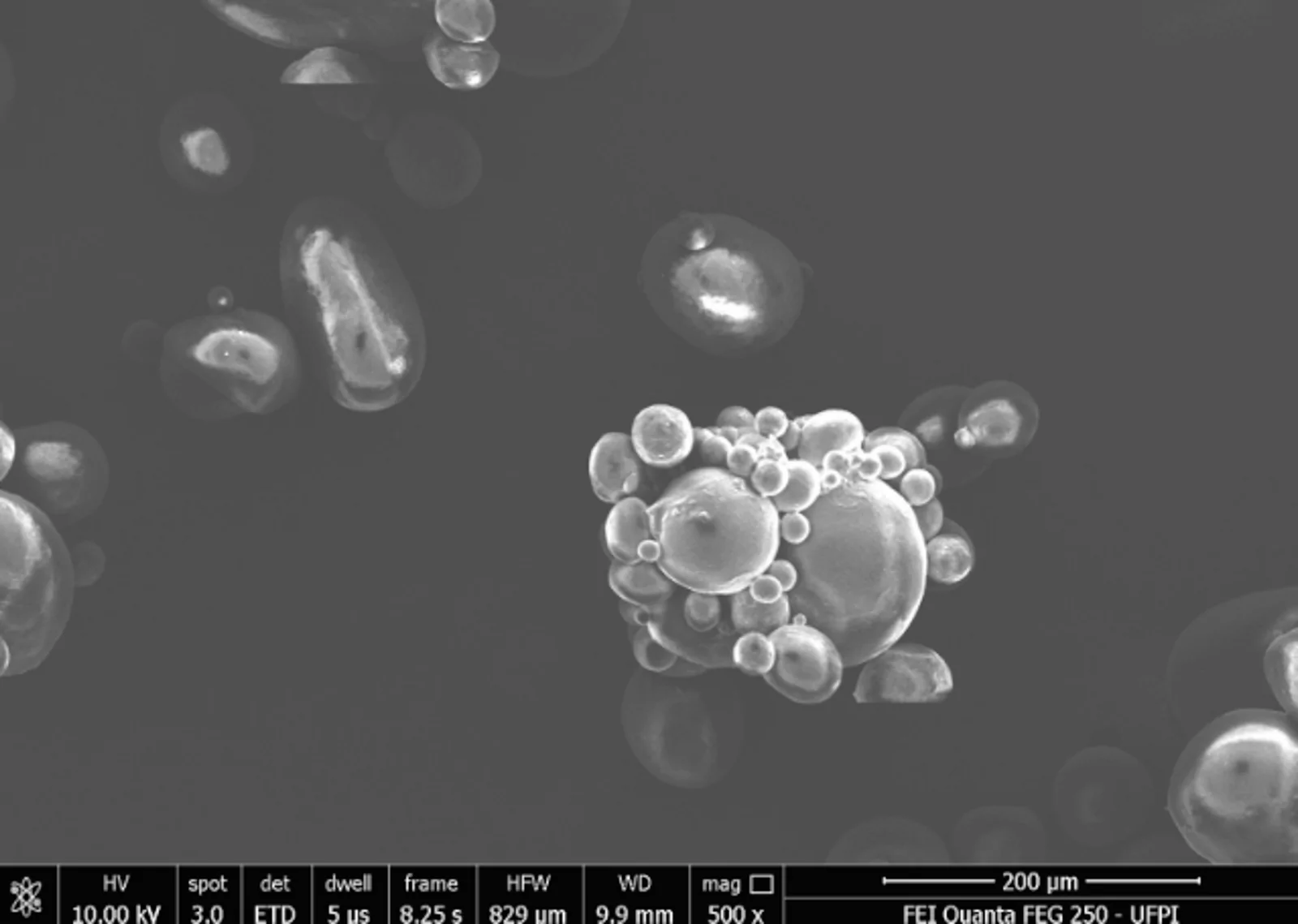
Scanning electron micrographs of flaxseed oil (*Linum usitatissimum*) microencapsulated into a carnauba wax lipidic matrix at 30%

Particle morphology was examined by scanning electron microscopy (SEM). The micrographs showed predominantly spherical particles with relatively smooth and well-defined external surfaces (Fig. 1). Although particle size varied within the sample, the particles generally exhibited a consistent spherical morphology, with diameters of approximately 200 μm based on the SEM scale.

Some larger particles exhibited smaller structures attached to their surfaces, resulting in localized particle aggregates. This surface organization was likely associated with wax solidification during cooling and may reflect partial particle coalescence or recrystallization of the lipid matrix. Overall, the micrographs indicated the formation of a continuous solid matrix surrounding the encapsulated material.

Smaller spherical structures associated with the surface of larger particles may have originated from secondary particle formation during emulsification or from wax recrystallization during cooling. The predominantly spherical morphology and continuous external matrix indicate successful particle formation by the melt-emulsification process. However, morphological characteristics alone do not demonstrate ruminal stability or controlled release; these properties require specific release or ruminal degradation measurements.

### Animals, experimental design, diets, and facilities

Fifty crossbred male lambs (Santa Inês × non-descript breed), approximately five months of age and with an initial body weight of 20.5 ± 3.0 kg, were allocated to five dietary treatments in a completely randomized design, with 10 animals per treatment. Lambs were housed individually in 2.0 × 2.0 m pens throughout the 70-day experimental period, which comprised 15 days of dietary adaptation followed by 55 days of data collection. Nutrient digestibility and nitrogen balance were assessed during two 5-day collection periods, conducted on days 20–25 and 40–45 of the experimental phase. During these assessments, lambs were transferred to individual metabolism crates for total collection of feces and urine.

The dietary treatments were: T1, control diet without supplemental lipid (CTRL); T2, 4% free flaxseed oil; T3, 8% free flaxseed oil; T4, 4% rumen-protected flaxseed oil (RPFO); and T5, 8% RPFO. Because RPFO contained flaxseed oil and carnauba wax at a fixed 30:70 ratio (w/w), inclusion of RPFO at 4% and 8% of dietary DM supplied 1.2% and 2.4% flaxseed oil, respectively. Diets containing 4% and 8% supplemental lipid material averaged 66 and 106 g ether extract/kg DM, respectively. Thus, at a given inclusion level, the amount of flaxseed oil supplied by RPFO was lower than that provided by the corresponding free-oil treatment because carnauba wax represented 70% of the protected product. This formulation was selected during development of the encapsulation system and achieved an encapsulation efficiency of 92.43%. Accordingly, the experimental design was intended to assess the nutritional responses to increasing inclusion of RPFO as a feed ingredient rather than to compare free and protected flaxseed oil at equivalent oil concentrations.

Before the experimental period, all lambs were individually weighed and subjected to the herd health protocol, which included vaccination against clostridial diseases (Resguard Multi^®^, Biovet, São Paulo, Brazil), anthelmintic treatment with 5% levamisole hydrochloride (Ripercol^®^, São Paulo, Brazil), and supplementation with vitamins A, D, and E.

Experimental diets contained forage and concentrate at a 40:60 ratio (DM basis) and were formulated according to NRC (2007) recommendations to support an average daily gain of 200 g. Maize silage was used as the forage source, whereas the concentrate included ground maize, soybean meal, the respective flaxseed oil source when applicable, and a mineral premix (Table 1). Diets were offered as total mixed rations twice daily at 08:00 and 16:00 h. Feed allowance was adjusted daily based on the amount of refusals from the previous day to maintain approximately 10% orts.

**Table 1:** Proportion of ingredients (g/kg) and chemical composition of the experimental diets

Item	Control	Free flaxseed oil		RP^3^-flaxseed oil	
	0%	4%	8%	4%	8%
Ingredients proportion (g/kg)					
Corn silage	400	400	400	400	400
Ground corn	417	372	322	372	322
Soybean meal	168	173	183	173	183
Free flaxseed oil	0	40	80	0	0
RP-flaxseed oil	0	0	0	40	80
Mineral mixture^1^	15	15	15	15	15
Chemical composition of the diet (g/kg DM)					
Dry matter (g/kg as fed)	631	635	640	635	640
Ash	52.7	52.3	52.2	52.3	52.2
Crude protein	146	144	144	144	144
Ether extract	29.0	67.5	106	65.3	104
Non-fibrous carbohydrates	486	455	421	455	421
_ a_NDFom^2^	286	281	276	281	276
Acid detergent fiber	131	129	128	129	128
Acid detergent lignin	29.3	28.9	28.4	28.9	28.4
Total digestible nutrients	760	800	839	800	839

^1^Assurance levels (per kilogram of active elements): 120 g of calcium, 87 g of phosphorus, 147 g of sodium, 18 g of sulfur, 590 mg of copper, 40 mg of cobalt, 20 mg of chromium, 1,800 mg of iron, 80 mg of iodine, 1,300 mg of manganese, 15 mg of selenium, 3,800 mg of zinc, 300 mg of molybdenum, and maximum 870 mg of fluoride

^2^Neutral detergent fiber assayed with a heat stable amylase and expressed exclusive of residual ash

^3^RP = Rumen-protected

#### Intake and growth performance

Individual feed intake was calculated daily as the difference between the amounts of feed offered and refused, with nutrient intake subsequently determined from the chemical composition of the offered diets and orts. Lambs were weighed after a 12-h period without access to feed at the beginning and end of the experiment. Total weight gain (TWG) was calculated as the difference between final and initial body weight, and average daily gain (ADG) was obtained by dividing TWG by the number of experimental days. Feed efficiency was calculated as the ratio of ADG to average daily dry matter intake.

### Ingestive behavior and diet particle selectivity

Ingestive behavior was monitored by two previously trained observers through direct visual observation at 5-min intervals over a continuous 24-h period, following the general approach described by Martin and Bateson (1993). Each observation was classified as feeding, ruminating, or idling according to predefined behavioral criteria. Before data collection, observers underwent standardized training and an inter-observer agreement assessment to ensure consistency in behavioral classification. Because the experimental conditions did not allow observers to be blinded to dietary treatments, all assessments were conducted using the same predefined ethogram to minimize observer-related bias. Artificial lighting was provided during nighttime observations to allow uninterrupted monitoring. Daily feeding, rumination, and idling times were expressed as min/d; feeding and rumination activities were also expressed relative to DM and aNDFom intake (min/kg).

Variables describing rumination activity were calculated from the observed behavioral data according to the procedures adapted from Beauchemin (2018). The number of ruminated boluses per day (NRB) was estimated from total daily rumination time and the mean time required to ruminate one bolus: NRB = TRT/TRB, where TRT is total rumination time (min/d) and TRB is the mean rumination time per bolus (min/bolus). Dry matter ruminated per bolus (DMB; g/bolus) was subsequently calculated as: DMB = DMI/NRB, where DMI represents daily dry matter intake (g/d). Additional variables related to chewing and rumination activity were derived from the corresponding observed times and bolus measurements.

Particle size distribution and feed sorting were assessed during the final week of the experiment. Samples of the total mixed ration (TMR) and corresponding orts were obtained 12 and 24 h after feed delivery on two consecutive days. After homogenization, representative subsamples were collected for DM determination at 105 °C for 24 h. Particle fractions were separated using the Penn State Particle Separator (PSPS), based on the procedures of Lammers et al. (1996) and Kononoff et al. (2003). The PSPS contained sieves with apertures of 19, 8, and 1.18 mm and a bottom pan for particles < 1.18 mm. Approximately 1 kg of fresh material was placed in the separator and subjected to eight sets of five horizontal movements. Material recovered from each fraction was weighed, and particle distribution was expressed on a DM basis.

For each particle fraction, the sorting index was calculated by dividing the observed intake of that fraction by its expected intake and multiplying the resulting value by 100. Expected intake was determined from the proportion of each particle fraction in the offered TMR and the total amount of feed consumed. An index of 100 indicated no sorting, whereas values > 100 represented preferential consumption and values < 100 indicated selective refusal of the respective fraction. The physical effectiveness factor was calculated as the proportion of DM retained above the 8-mm sieve (pef₈) or above the 1.18-mm sieve (pef₁.₁₈). Physically effective NDF (peNDF) was obtained by multiplying the NDF concentration of the diet by the corresponding pef.

#### Digestibility and nitrogen balance

Apparent nutrient digestibility and nitrogen balance were evaluated during two 5-day collection periods conducted on days 20–25 and 40–45 of the experimental phase. During each collection period, lambs were housed individually in metabolism crates (1.60 × 1.00 × 1.50 m) equipped with separate feeding and watering facilities and systems that allowed independent quantitative recovery of orts, feces, and urine.

Feed offered, orts, feces, and urine were quantitatively recorded over each 24-h collection interval. Urine was collected directly into containers containing 50 mL of 20% sulfuric acid to minimize nitrogen losses. At the end of each daily collection, total urinary volume was measured and a 10% aliquot was retained. Urine samples were maintained at pH < 3 and stored at − 20 °C until determination of urinary nitrogen concentration.

Apparent digestibility coefficients (ADC) were calculated from nutrient intake and fecal nutrient output as follows: ADC (g/kg) = [(nutrient intake − fecal nutrient output)/nutrient intake] × 1000. Nitrogen retention was calculated from the difference between nitrogen intake and nitrogen losses in feces and urine: N retention (g/d) = N intake − fecal N − urinary N.

### Blood parameters and analysis

Blood was collected on the first and last days of the experimental period at four time points relative to the morning feeding: immediately before feed delivery (0 h) and 2, 4, and 6 h thereafter, following the sampling schedule adapted from Huntington (1997). Samples were obtained by jugular venipuncture into 4-mL serum collection tubes (BD Vacutainer^®^, Becton, Dickinson and Co., Franklin Lakes, NJ, USA). After collection, blood samples were centrifuged at 2,500 rpm for 5 min, and the separated serum was transferred to 2-mL microtubes and stored until analysis.

Serum concentrations of albumin (ALB), triglycerides (TRI), creatinine (CRE), cholesterol (COL), total protein (TP), magnesium (Mg), and calcium (Ca), as well as the activities of aspartate aminotransferase (AST) and gamma-glutamyl transferase (GGT), were determined using commercial colorimetric assay kits (Labtest Diagnóstica S.A., Lagoa Santa, Minas Gerais, Brazil).

### Chemical composition and calculations

Ingredients, experimental diets, feed refusals, and fecal samples were dried in a forced-air oven at 55 °C for 72 h, ground through a 1 mm sieve, and analyzed for chemical composition. Dry matter (DM; AOAC Method 934.01), ash (AOAC Method 942.05), crude protein (CP; AOAC Method 954.01), and ether extract (EE; AOAC Method 920.39) were determined according to the procedures described by the Association of Official Analytical Chemists (1990). Neutral detergent fiber (NDF) and acid detergent fiber (ADF) were determined following the method of Van Soest et al. (1991), using filter bags as described by Senger et al. (2008), with the inclusion of thermostable α-amylase, and results expressed exclusive of residual ash. The NDF fraction was corrected for ash and protein (_a_NDFom) following Licitra et al. (1996). Non-fibrous carbohydrates (NFC) were obtained according to Hall (2007).

Total digestible nutrient (TDN) intake was estimated from the difference between nutrient intake and fecal nutrient output, using the equation: TDN intake (kg) = digestible CP + (2.25 × digestible EE) + digestible NFC + digestible NDF. The TDN concentration was then calculated as: TDN (g/kg DM) = (TDN intake/ DM intake) × 100. Total digestible nutrients (%TDN) were calculated as follows: TDN (%) = CPD% + NDFD% + NFCD% + (2.25 × EED%).

#### Statistical analyses

A completely randomized design was used, with five dietary treatments and 10 lambs per treatment. The individual animal was considered the experimental unit. Initial body weight was initially included in the statistical model as a covariate to account for potential baseline variation among animals. Because the covariate was not significant (*P* > 0.05) and its inclusion did not materially affect the estimated treatment effects, initial body weight was excluded from the final model. Dietary treatment was therefore included as the fixed effect in the final analysis.

Data were analyzed using the MIXED procedure of SAS 9.4 (SAS Institute Inc., Cary, NC, USA 2013). For intake, growth performance, and feed particle selectivity, the following model was used:$\:Yij\hspace{0.17em}=\mu\hspace{0.17em}+\hspace{0.17em}Ti\hspace{0.17em}+\hspace{0.17em}\varepsilon\:ij$. 

where *Yij* is the observed value of the response variable for animal *j* receiving treatment *i*, µ is the overall mean, *Ti* is the fixed effect of dietary treatment (*i* = 1, …, 5), and ε*ij* is the residual error associated with each observation.

For variables measured repeatedly over time, including ingestive behavior, nutrient digestibility, and nitrogen balance, data were analyzed using a repeated-measures approach. Evaluation day (for ingestive behavior) or collection period (for digestibility and nitrogen balance) was specified as the repeated factor within animal, with animal defined as the subject of the repeated measurements. The model included dietary treatment, time (evaluation day or collection period), and the treatment × time interaction as fixed effects. For ingestive behavior, observer was additionally included in the model to account for variability associated with behavioral assessment. The repeated-measures model was:

$$\:Yijk\hspace{0.17em}=\mu\hspace{0.17em}+\hspace{0.17em}Ti\hspace{0.17em}+\hspace{0.17em}Aj\left(i\right)\:+\:Timek\:+\:(T\:\times\:\:Time)ik\hspace{0.17em}+\hspace{0.17em}\varepsilon\:ijk$$

where *Y*ijk is the observed value of the response variable for animal *j* assigned to treatment *i* at time *k*; µ is the overall mean; *T*i is the fixed effect of dietary treatment (*i* = 1, …, 5); *A*j(i) is the effect of animal *j* nested within treatment *i* and represents the experimental subject on which repeated measurements were obtained; *Time*k is the fixed effect of time, corresponding to evaluation day for ingestive behavior or collection period for digestibility and nitrogen balance; (*T* × *Time*)ik is the interaction between dietary treatment and time; and εijk is the residual error associated with each observation. Because neither the effect of time nor the treatment × time interaction was significant (*P* > 0.05), observations obtained across evaluation days or collection periods were averaged within animal for presentation of treatment means.

Model residuals were examined to assess the assumptions underlying the statistical analyses and to identify potentially influential observations. Standardized residuals and Cook’s distance were used as diagnostic criteria. No missing observations or influential data points warranting exclusion were identified; therefore, all observations were retained in the analyses. Overall treatment effects were evaluated using the *F*-test, and when significant (*P* < 0.05), least-squares means were compared using Tukey’s adjustment for multiple comparisons. Statistical significance was declared at *P* < 0.05.

## Results

### Feed intake and growth performance

Dry matter intake (DMI) was significantly influenced by the inclusion of free or rumen-protected flaxseed oil (*P* = 0.009). Lambs fed the 8% free-oil diet exhibited the smallest DMI, whereas the 4% RPFO treatment resulted in the highest DMI (Table 2). Ash intake declined (*P* = 0.008) in animals receiving 8% free oil, while ether extract intake increased progressively (*P* < 0.001) with oil supplementation, reaching its maximum in the 8% RPFO group. Crude protein and _a_NDFom intakes decreased with 8% free oil, and diets control, 4% free oil and 8% RPFO inclusion presented higher intakes (*P* < 0.05) and feed efficiency (*P* = 0.012). Regarding NFC, higher intake occurred in animals fed with 4% RPFO and control group.

**Table 2:** Feed intake and performance growth of sheep fed diets containing free flaxseed oil and rumen-protected flaxseed oil (4% and 8%)

Variables	Control	Free flaxseed oil		RP^5^-flaxseed oil		SEM^1^	*P*-value^2^
	0%	4%	8%	4%	8%		
Intake (g/day)							
Dry matter	1595ab	1418b	1262c	1780a	1492b	0.06	0.009
Ash	80a	69ab	61b	57b	73ab	0.01	0.003
Crude protein	218a	197ab	175b	168b	206ab	0.01	0.008
Ether extract	46d	72c	66 cd	125b	153a	0.01	< 0.001
_ a_NDFom^3^	456a	398ab	348b	500a	411ab	0.04	0.003
NFC^5^	775ab	645b	531c	809a	628b	0.02	0.024
TDN^5^	1293	1161	995	1068	1284	0.07	0.314
Performance growth (kg)							
Initial BW	19.2	19.1	18.5	19.1	19.7	-	-
Final BW	33.1a	33.1a	27.3b	32.9a	33.4a	0.89	0.030
ADG (g/day)	253a	255a	160b	251a	249a	0.01	< 0.001
Feed efficiency^4^	0.14ab	0.16a	0.11c	0.12b	0.15a	0.02	0.012

^1^SEM: Standard error of the mean

^2^ Means with different letters in the row differ in the comparison by the Tukey test when *P* ≤ 0.05

^3^Neutral detergent fiber assayed with a heat stable amylase and expressed exclusive of residual ash

^4^Average Daily Gain (g)/ Dry Matter Intake (g)

^5^RP = Rumen-protected; NFC = non-fibrous carbohydrates; TDN = total digestible nutrients

Final body weight and average daily gain (ADG) was significantly affected (*P* < 0.05). Lambs fed 8% free oil showed the lowest final BW (27.3 kg) and ADG (169 g/day) comparable to other treatments.

### Ingestive behavior and particle selectivity

Rumination time differed among treatments (*P* = 0.004), with animals fed 8% free oil exhibiting reduced rumination compared with those receiving RPFO (Table 3); however, there was no difference compared to the control treatment. Eating and rumination efficiencies for DM and _a_NDFom in lambs fed RPFO were higher than with 8% free oil and RPFO inclusion (*P* < 0.001). Both the number of ruminated boluses (*P* = 0.022), and the amount of dry matter per bolus (*P* < 0.001) were higher in animals fed 4% RPFO (4 and 8% of inclusion) relative to those receiving 8% of free oil.

**Table 3:** Ingestive behavior of sheep fed diets containing free flaxseed oil and rumen-protected flaxseed oil (4% and 8%)

Variables	Control	Free flaxseed oil		RP^4^-flaxseed oil		SEM^1^	*P*-value^2^
	0%	4%	8%	4%	8%		
Time spent (min/day)							
Rumination	424ab	452a	336b	456a	468a	25.1	0.004
Eating	187	212	205	210	223	12.5	0.300
Idling	829ab	775ab	899a	774ab	749b	33.0	0.034
Efficiency rate (g/h)							
DM^4^ eating	512a	401b	369c	509a	401b	11.9	< 0.001
DM^4^ rumination	226a	188b	225a	234a	191ab	10.9	< 0.001
_ a_NDFom^3^ eating	146a	113a	102b	143a	111b	9.41	< 0.001
_ a_NDFom^3^ rumination	64.5a	52.8b	62.1a	65.8a	52.7b	5.25	< 0.001
Chewing							
Ruminal boli	666ab	730ab	559b	674ab	775a	45.2	0.022
Bolus rumination	56	53	55	60	55	2.61	0.447
g of DM per bolus	2.15ab	1.89b	1.73b	2.63a	2.58a	0.14	< 0.001

^1^SEM: Standard error of the mean

^2^ Means with different letters in the row differ in the comparison by the Tukey test when *P* ≤ 0.05

^3^Neutral detergent fiber assayed with a heat stable amylase and expressed exclusive of residual ash

^4^RPF = Rumen-protected; DM^4^ = dry matte

After 12 h of feeding (Table 4), the distribution of particles > 19 mm and > 8 mm was significantly affected by treatments (*P* = 0.002). Sheep fed 8% free oil selectively avoided larger particles, resulting in a higher proportion of feed residues on the bottom sieve (*P* < 0.001) compared with the control and RPFO treatments (4 and 8% inclusion). There were no differences between the other treatments. The physically effective factor (pef) and physically effective NDF (_pe_NDF) were greater in RPFO treatments (*P* ≤ 0.005), indicating better preservation of feed structure. After 24 h, however, no significant treatment effects were observed for most particle-size fractions (*P* > 0.300), except for the 1.8-mm fraction, which was lower in the free-oil treatment (*P* = 0.003).

**Table 4:** Selectivity of particles in the experimental diets containing free flaxseed oil and rumen-protected flaxseed oil (4% and 8%)

Particle distribution^5^	Control	Free flaxseed oil		RP^4^-flaxseed oil		SEM^1^	*P*-value^2^
	0%	4%	8%	4%	8%		
12 h after feeding (%)							
19 mm	27.2a	8.45bc	5.98c	19.2abc	22.1ab	3.47	< 0.001
8 mm	38.7a	30.1ab	15.8b	39.0a	40.0a	4.51	0.002
1.8 mm	14.0	16.3	11.8	14.0	14.3	1.92	0.603
Base	20.0b	45.2ab	66.4a	27.5b	23.6b	7.51	< 0.001
pef_8_	65.9a	38.5bc	21.7c	58.5ab	62.1ab	6.65	< 0.001
pef_1.18_	80.0a	54.8ab	33.6b	72.5a	76.4a	7.51	< 0.001
NDF^3^pef_8_	40.1a	26.0ab	15.1b	38.3a	40.5a	4.68	0.001
NDF^3^pef_1.18_	48.8a	36.8ab	23.1b	47.5a	49.9a	5.41	0.005
24 h after feeding (%)							
19 mm	21.6	13.2	23.0	16.9	20.0	3.95	0.425
8 mm	44.0	35.6	30.5	36.2	37.8	4.52	0.348
1.8 mm	14.4ab	15.2a	8.59b	15.1a	17.1a	1.462	0.003
Base	20.0	36.0	37.9	31.8	25.1	7.50	0.420
pef_8_	65.5	48.8	53.5	53.1	57.9	7.48	0.585
pef_1.18_	80.0	64.0	62.1	68.2	74.9	7.50	0.420
NDF^3^pef_8_	40.0	32.8	35.7	34.6	37.9	5.12	0.876
NDF^3^pef_1.18_	48.8	43.0	41.3	44.5	49.1	5.28	0.784

^1^SEM: Standard error of the mean

^2^ Means with different letters in the row differ in the comparison by the Tukey test when *P* ≤ 0.05

^3^Neutral detergent fiber assayed with a heat stable amylase and expressed exclusive of residual ash

^4^RP = Rumen-protected

^5^Diet particle size distribution was measured using the Penn State Particle Separator (Kononoff et al. 2003); pef8 and pef1.18 = physical effectiveness factor determined as the proportion of particles retained on 2 sieves (8 and 19 mm) (Lammers et al. 1996) and on 3 sieves (1.8, 8 and 19 mm) (Kononoff et al. 2003), respectively; NDFpef8 g and NDFpef1.18 h = effective neutral detergent fiber (NDF) of the sample multiplied by pef8 and pef1.18, respectively

### Nutrient digestibility and nitrogen (N) balance

Overall, the inclusion of dietary oil negatively affected nutrient digestibility. Digestibility coefficients were highest in the control group and progressively declined with RPFO supplementation, reaching the lowest values at the 4% inclusion level (*P* < 0.05). Dry matter and ash digestibility decreased (*P* ≤ 0.005) in lambs fed RPFO diets compared with the control diet (Table 5). In contrast, EE digestibility increased markedly (*P* < 0.001) with higher oil inclusion, showing the greatest improvement at 8% free oil and RPFO. Crude protein digestibility also differed among treatments (*P* = 0.013), with the highest value observed in animals receiving 8% compared to 4% RPFO, and with no differences compared to other treatments. In contrast, total carbohydrate digestibility was lower in diets with RPF, regardless of the level (*P* < 0.001). The TDN content of the diets was highest (*P* = 0.002) in the 8% oil-free and RPFO groups compared to 4% RPFO, and there were no differences compared to other treatments.

**Table 5:** Digestibility of nutrients in sheep fed diets containing free flaxseed oil and rumen-protected flaxseed oil (4% and 8%)

Digestibility (g/kg of ingested)	Control	Free flaxseed oil		RP^4^-flaxseed oil		SEM^1^	*P*-value^2^
	0%	4%	8%	4%	8%		
Dry matter	811a	789ab	801ab	761b	766b	10.1	0.005
Ash	558a	475ab	413b	392b	434b	21.4	< 0.001
Crude protein	789ab	777ab	798ab	750b	811a	11.9	0.013
Ether extract	761d	813 cd	923a	841bc	914ab	19.2	< 0.001
_a_NDFom^3^	705a	653ab	543ab	505b	562ab	47.4	0.030
NFC^4^	970	963	965	999	973	11.4	0.181
TDN^4^	810ab	818ab	872a	789b	862a	15.3	0.002

^1^SEM: Standard error of the mean

^2^ Means with different letters in the row differ in the comparison by the Tukey test when *P* ≤ 0.05

^3^Neutral detergent fiber assayed with a heat stable amylase and expressed exclusive of residual ash

^4^RP = Rumen-protected; NFC = Non-fibrous carbohydrates; TDN = total digestible nutrients

Nitrogen (N) intake (*P* = 0.008), N digested (*P* = 0.005), and N retained (*P* = 0.003) were lower in lambs fed 4% RPFO (Table 6) with no differences compared to other treatments. Urinary and total N excretion were significantly affected by treatments (*P* < 0.001), with lower excretion values recorded in animals receiving 8% free oil. In contrast, the ratio of excreted-to-intake N was higher and retained-to-intake and retained-to-digested N was lower (*P* < 0.001) compared to lambs receiving 8% free oil and control diets (*P* < 0.001).

**Table 6:** Nitrogen (N) balance in sheep fed diets containing free flaxseed oil and rumen-protected flaxseed oil (4% and 8%)

Nitrogen balance (g/d)	Control	Free flaxseed oil		RP^4^-flaxseed oil		SEM^1^	*P*-value^2^
	0%	4%	8%	4%	8%		
N Intake	35.0a	31.6ab	27.0b	28.1b	33.0ab	1.64	0.008
N Fecal	7.27	6.95	5.47	6.91	6.26	0.496	0.105
N Urinary	4.30bc	4.90abc	3.15c	5.70ab	6.86a	0.49	< 0.001
N Excreted	11.6a	11.9a	8.63b	12.6a	13.1a	0.64	< 0.001
N Digested	27.8a	24.6ab	21.5b	21.2b	26.8ab	1.41	0.005
N Retained	23.5a	19.7ab	18.4ab	15.5b	19.9ab	1.30	0.003
N Excreted/N Intake	33.3c	37.5bc	32.7c	44.7a	40.3ab	1.68	< 0.001
N Retained/N Intake	66.7a	62.5ab	67.3a	55.3c	59.7bc	1.68	< 0.0001
N Retained/N Digested	84.4a	80.8ab	84.1a	73.2b	73.7b	2.05	< 0.001

^1^SEM: Standard error of the mean

^2^ Means with different letters in the row differ in the comparison by the Tukey test when *P* ≤ 0.05

^3^Neutral detergent fiber assayed with a heat stable amylase and expressed exclusive of residual ash

^4^RP = Rumen-protected

### Blood metabolites

No differences (*P* > 0.05) were observed among treatments for total protein, albumin, AST, cholesterol, creatinine, GGT, calcium, or magnesium concentrations, indicating that hepatic, and renal function remained within normal physiological ranges across all diets (Table 7). Triglycerides (TGL) concentrations, however, were influenced by treatments (*P* = 0.010), with higher levels observed in lambs fed 8% free oil compared with those receiving RPFO or the control diet. Across the experimental period (Table 8), significant increases were detected in albumin, total protein, TGL, cholesterol, and calcium concentrations (*P* < 0.001), whereas creatinine and magnesium declined (*P* ≤ 0.010). No changes in AST or GGT activities were observed (*P* > 0.05), confirming that none of the dietary treatments induced hepatic dysfunction during the feeding period. No treatment × time interactions were detected (*P* > 0.05). Within each treatment, serum urea concentrations did not differ between the initial and final sampling days. However, across treatments, numerical increases in final urea levels were noted, particularly in the 4% and 8% free-oil groups (Table 9).

**Table 7:** Blood parameters of lambs fed diets containing free flaxseed oil and rumen-protected fat from flaxseed oil (4% and 8%)

Blood	Control	Free flaxseed oil		RP^4^-flaxseed oil		SEM^1^	*P*-value^2^
	0%	4%	8%	4%	8%		
Albumin (g/dL)	2.84	2.95	2.93	2.94	2.87	0.092	0.875
AST^4^ (U/L)	75.5	91.1	98.5	78.1	85.1	8.52	0.201
Cholesterol mg/dL)	44.3	45.2	45.0	46.3	48.7	2.83	0.765
Creatinine (mg/dL)	0.61	0.68	0.71	0.63	0.64	0.031	0.111
GGT^4^ (U/L)	40.8	45.0	41.9	40.4	43.3	2.70	0.667
Total protein (g/dL)	5.75	5.95	6.48	6.20	6.21	0.203	0.056
Triglycerides (mg/dL)	19.2ab	17.6b	25.1a	16.3b	18.9ab	2.07	0.010
Mg (mmol/L)	1.19	1.29	1.30	1.30	1.15	0.082	0.423
Ca (mg/dl)	8.05	9.43	7.95	8.83	8.17	0.633	0.302

^1^SEM: Standard error of the mean

^2^Means with different letters in the row differ in the comparison by the Tukey test when *P* ≤ 0.05

^4^RP = Rumen-protected; Aspartate aminotransferase (AST), and gamma-glutamyl transferase (GGT)

**Table 8:** Comparison between initial (1st) and final (55th) blood sampling collection days

Blood variables	Initial	Final	SEM^1^	*P*-value^2^
Albumin (g/dL)	2.63	3.18	0.05	< 0.001
AST^3^ (U/L)	84.8	86.6	5.02	0.788
Cholesterol mg/dL)	32.8	59.1	1.67	< 0.000
Creatinine (mg/dL)	0.72	0.59	0.02	< 0.000
GGT^3^ (U/L)	40.2	44.3	1.59	0.060
Total protein (g/dL)	5.64	6.59	0.12	< 0.001
Triglycerides (mg/dL)	13.9	25.0	1.22	< 0.001
Mg (mmol/L)	1.33	1.16	0.05	0.010
Ca (mg/dL)	7.12	9.85	0.37	< 0.001

^1^SEM: Standard error of the mean

^2^Means differ statistically between the initial and final sampling by the Tukey test when *P* ≤ 0.05

^3^Aspartate aminotransferase (AST), and gamma-glutamyl transferase (GGT)

**Table 9:** Interaction effect on serum urea concentration at different sampling times

Sampling	Control	Free flaxseed oil		RP^1^-flaxseed oil	
	0%	4%	8%	4%	8%
Initial	32.28Aa	19.8Ab	23.6Ab	28.6Aa	22.1Aa
Final	34.6Aa	41.8Aa	42.6Aa	30.7Aa	33.5Aa

*Means with different uppercase letters indicate statistically significant differences among treatments (*P* ≤ 0.05)

*Means with different lowercase letters indicate significant differences between the initial and final sampling (*P* ≤ 0.05)

^1^RP = Rumen-protected

## Discussion

The high encapsulation yield (92%) and efficiency (94%) confirmed the structural integrity of the carnauba-wax matrix (Fig. 1), supporting its suitability as a RPFO system. These values exceed those reported by Lima et al. (2023) and Feitosa et al. (2024), who obtained 85% and 61.3% for yield, and 92% and 75% for efficiency, respectively, indicating strong oil retention capacity. Differences among studies may be partially explained by fatty acid composition, as oils richer in polyunsaturated fatty acids (PUFA), such as flaxseed oil, are more susceptible to ruminal biohydrogenation if not effectively protected (Diogénes et al. 2020; Amin et al. 2021; Sun et al. 2021). As described in the Materials and Methods, the RPFO was produced using a fixed 30:70 oil-to-carnauba wax ratio established during the optimization of the encapsulation process. Therefore, differences in dietary flaxseed oil concentration reflect the composition of the encapsulated ingredient rather than inconsistencies in diet formulation.

The lower DMI observed with 8% free oil confirms the known inhibitory effects of high levels of UFA on rumen microbial activity and fiber digestion (Jenkins 1993; Bauman and Griinari 2003; Jenkins et al. 2008). Reduced crude protein and _a_NDFom intake in this treatment reflects both decreased voluntary intake and increased feed sorting. In contrast, the 4% RPFO treatment maintained the highest DMI, suggesting that encapsulation mitigated the negative effects of free PUFA on ruminal fermentation, as previously described for protected lipids (Soares et al. 2022; Netto et al. 2022; Lima et al. 2023).

Lambs fed the diet containing 8% free oil exhibited an 18% lower final body weight and an 8% lower average daily gain (ADG) than those receiving the other treatments, indicating that excessive dietary inclusion of free lipids may have impaired nutrient utilization efficiency. This is consistent with reports that high PUFA levels suppress fibrolytic populations and reduce microbial protein synthesis (Lourenço et al. 2010; Ferreira et al. 2016). Conversely, RPFO maintained performance comparable to the control, supporting the concept that protected lipids can increase dietary energy density without markedly depressing intake or growth (Ferreira et al. 2020; Souza et al. 2025).

The reduced rumination time and fiber efficiency observed with 8% free oil reinforce the disruptive effect of unprotected lipids on rumen mat structure and microbial adhesion to fiber (Mertens 1993; Schulze et al. 2014). The greater number of ruminated boluses and higher DM per bolus in RPFO-fed lambs indicate improved chewing efficiency and preservation of physically effective fiber, consistent with Mertens (1997). Particle selectivity data further support this interpretation: sheep fed 8% free oil rejected larger particles after 12 h, while RPFO maintained more uniform particle distribution and higher pef and peNDF values, indicating better structural preservation of the diet (Beauchemin et al. 2018).

Digestibility responses demonstrate the complex interaction between lipid supplementation and rumen fermentation. As expected, ether extract digestibility increased with free oil inclusion, particularly at 8%, reflecting greater lipid availability for post-ruminal absorption (Harvatine and Allen 2006; Bhatt et al. 2017). However, DM, ash, and total carbohydrate digestibility declined in RPFO treatments, especially at 4%, indicating that even protected lipids may partially interfere with fiber degradation (Parente et al. 2020; Yang et al. 2022). These results align with Jenkins (1993), who described the sensitivity of cellulolytic bacteria to long-chain UFA.

Nitrogen balance results confirm that 4% RPFO reduced N intake, digested N, and retained N, while 8% free oil reduced ADG but showed lower urinary N excretion. The higher excreted-to-intake N ratio with 4% RPFO indicates reduced nitrogen utilization efficiency compared with the control and 8% free oil treatments. The impaired nitrogen retention observed with excessive lipid inclusion may be associated with altered ruminal microbial activity and reduced synchronization between energy and nitrogen availability (Bionaz et al. 2020; Bezerra et al. 2026). Although some studies report improved N efficiency with protected fats (Feitosa et al. 2024), the present results indicate that this response is dose-dependent and influenced by intake patterns (INCT 2024).

Blood metabolites confirmed the physiological safety of lipid supplementation. The absence of changes in AST, GGT, creatinine, and albumin indicates preserved hepatic and renal function (Kaneko et al. 2008). The increase in triglycerides with 8% free oil reflects greater immediate lipid absorption, whereas RPFO maintained more stable triglyceride concentrations, likely due to slower intestinal release and absorption of encapsulated lipids (Toral et al. 2024; Perkins et al. 2025). These findings suggest that protected lipids moderate postprandial lipid flux without inducing metabolic disturbances.

Overall, the results demonstrate that excessive inclusion of free flaxseed oil (8%) compromises intake, rumination efficiency, and growth performance due to ruminal disruption. Rumen-protected flaxseed oil partially mitigated these negative effects, maintaining intake and behavioral parameters, although some reductions in digestibility and nitrogen efficiency were observed. These findings highlight the need to carefully optimize inclusion levels to balance ruminal stability and post-ruminal energy supply.

Because the primary objective of this study was to assess animal performance and nutrient utilization, no assays were conducted to quantify the ruminal release rate or bypass efficiency of the encapsulated flaxseed oil. As such, direct in situ or in vivo evidence of wax-matrix stability and post-ruminal lipid availability is not available. Although the performance and nitrogen balance results suggest partial post-ruminal absorption, future studies should incorporate in situ or in vitro rumen incubation-fermentation, or post-ruminal flow markers to validate the release kinetics of carnauba-wax-based protected lipids.

The RPFO contained approximately 70% carnauba wax and 30% flaxseed oil. Achieving equal oil concentrations across diets would have required altering the matrix’s structural composition, thereby compromising its protective integrity. Therefore, dietary treatments were formulated according to the inclusion level of the rumen-protected ingredient, since the encapsulation process required a fixed oil-to-carnauba wax ratio (30:70, w/w) previously optimized to maximize encapsulation efficiency and particle stability. Consequently, differences in flaxseed oil concentration among treatments were an intrinsic characteristic of the encapsulated product rather than a consequence of diet formulation.

We acknowledge that this results in differing actual oil intakes between protected and unprotected treatments, which may affect digestibility and metabolic responses. This limitation is now explicitly recognized in the manuscript. In this context, the increase in ruminal EE digestibility and nitrogen utilization as RPFO inclusion rose from 4% to 8% may reflect partial matrix rupture or diminished protection efficiency at higher inclusion levels. These findings indicate that the encapsulation system may not maintain complete structural stability under elevated dietary loading, underscoring the need for optimization and targeted validation.

## Conclusion

Supplementation with 8% free flaxseed oil impaired dry matter intake, rumination efficiency, and average daily gain, confirming the adverse effects of high levels of unprotected PUFA on ruminal function, whereas RPFO provided a safer alternative without compromising hepatic or renal function. Although lipid supplementation increased ether extract digestibility, reductions in nutrient digestibility and nitrogen utilization indicate that RPFO inclusion should be carefully optimized to preserve ruminal stability and nutrient-use efficiency. Further studies should establish optimal inclusion levels, assess potential effects on ruminal fermentation and post-ruminal lipid utilization, and evaluate the long-term productive, environmental, and economic feasibility of RPFO under commercial lamb-production conditions.

## Data Availability

Data will be made available on reasonable request.

## References

[CR2] Amin N, Tagliapietra F, Arango S, Guzzo N, Bailoni L (2021) Free and microencapsulated essential oils incubated in vitro: ruminal stability and fermentation parameters. Animals 11:180. 10.3390/ani11010180 PMC7828777 33466658

[CR3] Association of Official Analytical Chemists (1990) Official methods of analysis, 15th edn. AOAC, Washington DC

[CR4] Bas P, Berthelot V, Pottier E, Normand J (2007) Effect of level of linseed on fatty acid composition of muscles and adipose tissues of lambs with emphasis on trans fatty acids. Meat Sci 77:678–688. 10.1016/j.meatsci.2007.05.022 22061958

[CR5] Bauman DE, Griinari JM (2003) Nutritional regulation of milk fat synthesis. Annu Rev Nutr 23:203–227. 10.1146/annurev.nutr.23.011702.073408 12626693

[CR6] Beauchemin KA (2018) Current perspectives on eating and rumination activity in dairy cows. J Dairy Sci 101:4762–4784. 10.3168/jds.2017-13706 29627250

[CR7] Bezerra LR, Mazza PHS, Lucena KHOS, Amorim MAF, Moura JFP, Oliveira JPF, Oliveira RL, Barbosa AM, Fonseca MA, Pereira Filho JM (2025) Slow-release urea technologies: effects on nitrogen utilization and small ruminant performance. Ruminants 5:50. 10.3390/ruminants5040050

[CR8] Bezerra LR, Oliveira JPF, Vaz AFM, Lucena KHOS, Barros LS, Fortunato YMA, Gonzaga Neto S, Pereira ES, Oliveira RL, Pereira Filho JM (2026) Nitrogen source–carbohydrate synchronization in ruminant nutrition: a systematic review. Animals 16:239. 10.3390/ani16020239 PMC12838081 41594428

[CR9] Bhatt RS, Sahoo A, Soni LK, Gadekar Y (2017) Effect of protected fat as Ca-soap and formaldehyde-treated soybean in lamb diets. Agricultural Res 6:427–435. 10.1007/s40003-017-0273-7

[CR10] Bionaz M, Vargas-Bello-Pérez E, Busato S (2020) Advances in fatty acids nutrition in dairy cows: from gut to cells and effects on performance. J Anim Sci Biotechnol 11:110. 10.1186/s40104-020-00512-8 PMC7667790 33292523

[CR11] Carvalho ADB, Silva AL, Silva ADA, Netto AJ, Medeiros TTB, Araújo Filho JM, Agostini DLS, Oliveira DLV, Mazzetto SE, Kotzebue LRV, Oliveira JR, Oliveira RL, Bezerra LR (2019) Effect of slow-release urea microencapsulated in beeswax in ruminant diets. Small Ruminant Res 179:56–63. 10.1016/j.smallrumres.2019.09.005

[CR12] de Freitas CAS, Sousa PHM, Soares DJ, Silva JYG, Benjamin SR, Guedes MIF (2019) Carnauba wax uses in food: a review. Food Chem 291:38–48. 10.1016/j.foodchem.2019.03.133 31006469

[CR13] de Lucena KHOS, Mazza PH, Oliveira RL, Barbosa AM, Pereira Filho JM, Bessa RJB, Alves SP, Edvan RL, Pereira ES, Fonseca M, Silva Filho EC, Bezerra LR (2024) Slow-releasing urea coated with vegetable lipids: effects on lamb performance. Anim Feed Sci Technol 310:115925. 10.1016/j.anifeedsci.2024.115925

[CR14] de Medeiros IG, Carvalho GGP, Oliveira RL, Ribeiro OL, Costa RM, Silva RR (2019) Carnauba wax as a wall material for urea microencapsulation. J Anim Physiol Anim Nutr 103:1475–1486. 10.1002/jsfa.9275

[CR15] Dewanckele L, Toral PG, Vlaeminck B, Fievez V (2020) Role of rumen biohydrogenation intermediates and rumen microbes in diet-induced milk fat depression: an update. J Dairy Sci 103:7655–7681. 10.3168/jds.2019-17662 32600765

[CR16] Diogénes L, Bezerra L, Pereira Filho J, Silva Junior J, Oliveira J, Moura J, Barbosa A, Souza M, Sousa S, Pereira E, Oliveira R (2020) Effects of dietary inclusion of buriti oil on lamb performance, carcass traits, digestibility, nitrogen balance, ingestive behavior and blood metabolites. Animals 10: 1973. 10.3390/ani10111973 PMC7693342 33126402

[CR17] Feitosa T, Bezerra L, Silva AC, Oliveira D, Mazzetto S, Cavalcanti M, Pereira Filho JM, Oliveira R, Oliveira J, Fonseca M, Silva A (2024) Manufacturing technology for rumen-protected fat using beeswax. Trop Anim Health Prod 57:6. 10.1007/s11250-024-04261-y 39710808

[CR19] Ferreira EM, Pires AV, Susin I, Gentil RS, Parente MOM, Nolli CP, Meneghini RCM, Mendes CQ, Ribeiro CVDM (2016) Nutrient digestibility and ruminal fatty acid metabolism in lambs supplemented with soybean oil partially replaced by fish oil blend. Anim Feed Sci Technol 216:30–39. 10.1016/j.anifeedsci.2015.09.007

[CR18] Ferreira AMS, Silva SP, Faria CU, Mendes EDM, Felipe EF (2020) Consumption of dry matter observed and predicted by nutritional systems in Senepol bulls kept in confinement. Bioscience J 36:2165–2171. 10.14393/BJ-v36n6a2020-48025

[CR20] Hall MB (2007) Methodological challenges in carbohydrate analyses. Revista Brasileira de Zootecnia 36:359–367. 10.1590/S1516-35982007001000032

[CR21] Harvatine KJ, Allen MS (2006) Fat supplements affect fractional rates of ruminal fatty acid biohydrogenation and passage in dairy cows. J Nutr 136:677–685. 10.1093/jn/136.3.677 16484542

[CR22] Hess BW, Moss GE, Rule DC (2008) A decade of developments in fat supplementation research with beef cattle and sheep. J Anim Sci 86:E188–E204. 10.2527/jas.2007-0546 18156350

[CR23] Hiller B (2014) Recent developments in lipid metabolism in ruminants: the role of fat in maintaining animal health and performance. Anim Prod Sci 54:1549–1560. 10.1071/AN14555

[CR24] Huntington GB (1997) Starch utilization by ruminants: From basics to the bunk. J Anim Sci 75(3):852–867 10.2527/1997.753852x 9078506

[CR25] INCT Animal Science (2024) Nutritional requirements of goats and sheep: BR-Caprinos & Ovinos. Scienza Publishing, Viçosa

[CR26] Jenkins TC (1993) Lipid metabolism in the rumen. J Dairy Sci 76:3851–3863. 10.3168/jds.S0022-0302(93)77727-9 8132891

[CR27] Jenkins TC, Wallace RJ, Moate PJ, Mosley EE (2008) Recent advances in biohydrogenation of unsaturated fatty acids within the rumen microbial ecosystem. J Anim Sci 86:397–412. 10.2527/jas.2007-0588 18042812

[CR28] Kaneko JJ, Harvey JW, Bruss ML (2008) Clinical biochemistry of domestic animals, 6th edn. Academic, San Diego

[CR29] Kononoff PJ, Heinrichs AJ, Buckmaster DR (2003) Modification of the Penn State forage and total mixed ration particle separator and the effects of moisture content on its measurements. J Dairy Sci 86:1858–1863. 10.3168/jds.S0022-0302(03)73773-4 12778598

[CR30] Lammers BP, Buckmaster DR, Heinrichs AJ (1996) Method for particle size analysis of rations. J Dairy Sci 79:922–928. 10.3168/jds.S0022-0302(96)76442-1 8792291

[CR31] Licitra G, Hernandez TM, Van Soest PJ (1996) Standardization of nitrogen fractionation procedures. Anim Feed Sci Technol 57:347–358. 10.1016/0377-8401(95)00837-3

[CR32] Lima JAM, Bezerra LR, Feitosa TJO, Oliveira J, Oliveira DLV, Mazetto SE, Cavalcante MT, Pereira Filho JM, Oliveira RL, Oliveira JPF, Silva AL (2023) Rumen-protected fat and ruminal function in sheep. Trop Anim Health Prod 55:142. 10.1007/s11250-023-03563-x 37016138

[CR33] Lourenço M, Ramos-Morales E, Wallace RJ (2010) The role of microbes in rumen lipolysis and biohydrogenation and their manipulation. Animal 4:1008–1023. 10.1017/S175173111000042X 22444606

[CR34] Martin P, Bateson P (1993) Measuring behaviour, 2nd edn. Cambridge University Press, Cambridge

[CR36] Mertens DR (1993) Kinetics of cell wall digestion and passage in ruminants. In: Jung HG, Buxton DR, Hatfield RD, Ralph J (eds) Forage cell wall structure and digestibility. 10.2134/1993.foragecellwall.c21

[CR35] Mertens DR (1997) Creating a system for meeting the fiber requirements of dairy cows. J Dairy Sci 80:1463–1481. 10.3168/jds.S0022-0302(97)76075-2 9241608

[CR38] National Research Council (2007) Nutrient requirements of small ruminants: sheep, goats, cervids, and New World camelids. National Academies, Washington DC. 10.17226/11654

[CR37] Netto AJ, Azevedo Silva AM, Bezerra LR, Barros Carvalho A, Silva Agostini DL, Vasconcelos de Oliveira DL, Mazzetto SE, Viana Kotzebue LR, Ribeiro Oliveira J, Oliveira RL, Pereira Filho JM, Silva AL (2022) Lipid microspheres containing urea for slow release of non-protein nitrogen in ruminant diets. Anim Prod Sci 62:191–200. 10.1071/AN20694

[CR39] Parente MOM, Rocha KS, Bessa RJB, Parente HN, Zanine AM, Machado NAF, Lorenco Junior JB, Bezerra LR, Landim AV, Alves SP (2020) Effects of dietary inclusion of babaçu oil or buriti oil on lamb performance, meat quality and fatty acid composition. Meat Sci 160:107971. 10.1016/j.meatsci.2019.107971 31669864

[CR40] Perkins LS, Nugent AP, Woodside JV, Cardwell CR, Scollan ND (2025) Consuming grass-finished lamb improves blood plasma omega-3 fatty acid response among healthy consumers. Eur J Nutr 64:242. 10.1007/s00394-025-03765-z PMC12279616 40690019

[CR42] SAS Institute Inc (2013) SAS/STAT 9.4 user’s guide. SAS Institute Inc

[CR44] Schulze AKS, Weisbjerg MR, Nørgaard P (2014) Effects of feeding level and neutral detergent fiber content of grass-clover silages on chewing activity, fecal particle size and digestibility in dairy heifers. Animal 8:1945–1954. 10.1017/S1751731114002055 25209214

[CR43] Senger CC, Kozloski GV, Sanchez LMB, Mesquita FR, Alves TP, Castagnino DS (2008) Evaluation of autoclave procedures for fibre analysis in forage and concentrate feedstuffs. Anim Feed Sci Technol 146:169–174. 10.1016/j.anifeedsci.2007.12.008

[CR45] Silva A, Pereira Filho JM, Oliveira J, Lucena K, Mazza P, Silva Filho E, Nascimento A, Pereira E, Vaz A, Barbosa A, Oliveira R, Bezerra L (2023) Effect of slow-release urea on intake, ingestive behavior, digestibility, nitrogen metabolism, microbial protein production, and blood and ruminal parameters of sheep. Trop Anim Health Prod 55:414. 10.1007/s11250-023-03833-8 37996715

[CR46] Silva EB, Kavamura VN, Freitas FMM, Souza AJ, Pereira APA (2025) Current knowledge of carnauba plant (Copernicia prunifera): current stage, trends, and future perspectives. Environments 12:437. 10.3390/environments12110437

[CR48] Soares RL, Queiroga RCRE, Bessa RJB, Sousa FA, Fernandes BDO, Souza AP, Medeiros AN (2022) Performance and carcass characteristics of lambs fed diets containing different types of carbohydrates associated with polyunsaturated fatty acids. Acta Scientiarum Anim Sci 44:e56131. 10.4025/actascianimsci.v44i1.56131

[CR49] Souza R, Alcalde CR, Damasceno JC, Passetti LCG, Molina BSL, Hygino B, Horst JA, Santos GT (2025) Impact of doses of protected fat supplementation on milk composition and performance of lactating Saanen goats. Semina: Ciências Agrárias 46:739–756. 10.5433/1679-0359.2025v46n3p739

[CR50] Sun CH, Lee JS, Nejad JG, Kim WS, Lee HG (2021) Effect of a rumen-protected microencapsulated supplement from linseed oil on growth performance, meat quality, and fatty acid composition in Korean native steers. Animals 11:1253. 10.3390/ani11051253 PMC8145495 33925315

[CR51] Toral PG, Hervás G, Frutos P (2024) Research on ruminal biohydrogenation: achievements, gaps in knowledge, and future approaches from the perspective of dairy science. J Dairy Sci 107:10115–10140. 10.3168/jds.2023-24591 39154717

[CR52] Van Soest PJ, Robertson JB, Lewis BA (1991) Methods for dietary fiber, neutral detergent fiber, and nonstarch polysaccharides in relation to animal nutrition. J Dairy Sci 74:3583–3597. 10.3168/jds.S0022-0302(91)78551-2 1660498

[CR53] Wardiman B, Natsir A, Syahrir S (2024) Bibliometric analysis of trends and future research directions in protected fat supplementation in ruminant nutrition. Int J Des Nat Ecodyn 19:1591–1602. 10.18280/ijdne.190513

[CR54] Yang C, Deng X, Lund P, Liu H, Ding X, Fu Z, Zhang N, Li J, Dong L (2022) Rumen microbiota-host transcriptome interaction mediates the protective effects of trans-10, cis-12 CLA on facilitating weaning transition of lambs. Anim Nutr 12:345–359. 10.1016/j.aninu.2022.11.002 PMC9898626 36788929

